# Supplementary material for: Homophilic ATP1A1 binding induces activin A secretion to promote EMT of tumor cells and myofibroblast activation
Source: Nat Commun. 2022 May 26;13:2945. doi: 10.1038/s41467-022-30638-4 (PMC9135720; doi:10.1038/s41467-022-30638-4)
Supplement: Supplementary file 1 — Supplementary Information [file 41467_2022_30638_MOESM1_ESM.pdf]

Supplementary information for

Homophilic ATP1A1 Binding Induces Activin A Secretion to Promote EMT of Tumor Cells and Myofibroblast Activation

Yi-Ing Chen, Chin-Chun Chang, Min-Fen Hsu, Yung-Ming Jeng, Yu-Wen Tien, Ming-Chu Chang, Yu-Ting Chang, Chun-Mei Hu*, and Wen-Hwa Lee*

Correspondence to: whlee@uci.edu and CMHU1220@sinica.edu.tw

This document includes:

Table S1
Figures S1 to S7

Supplemental Table 1. Primer sequences for real-time PCR

Gene	Forward	Reverse
**CLDN1**	5’-CAGCTGTTGGGCTTCATTCTC-3’	5’-ATCACTCCCAGGAGGATGCC-3’
**VIM**	5’-GTTTCCAAGCCTGACCTCAC-3’	5’-GCTTCAACGGCAAAGTTCTC-3’
**Snail**	5’-TTGGGCCAACTTCCCAAGCA-3’	5’-AGAGAAGGCCTTTCCACAGGT-3’
**Twist1**	5’-GCAGGACGTGTCCAGCTC-3’	5’-CTGGCTCTTCCTCGCTGTT-3’
**ZEB1**	5’-GCACCTGAAGAGGACCAGAG-3’	5’-TGCATCTGGTGTTCCATTTT-3’
**ZEB2**	5’-AAGGAGCAGGTAATCGCAAG-3’	5’-GGAACCAGAATGGGAGAAACG-3’
**ACTA2**	5’-GAGCGTGGCTATTCCTTCGT-3’	5’-TTCAAAGTCCAGAGCTACATAACACAGT-3’
**COL1A1**	5’-CTGCTGGACGTCCTGGTGAA-3’	5’-ACGCTGTCCAGCAATACCTTGAG-3’
**INHBA**	5’-GCAGTCTGAAGACCACCCTC-3’	5’-ATGATCCAGTCATTCCAGCC-3’
**GAPDH**	5’-GGCTCTCCAGAACATCATCCCTGC-3’	5’-GGGTGTCGCTGTTGAAGTCAGAGG-3’

Supplemental Figure 1

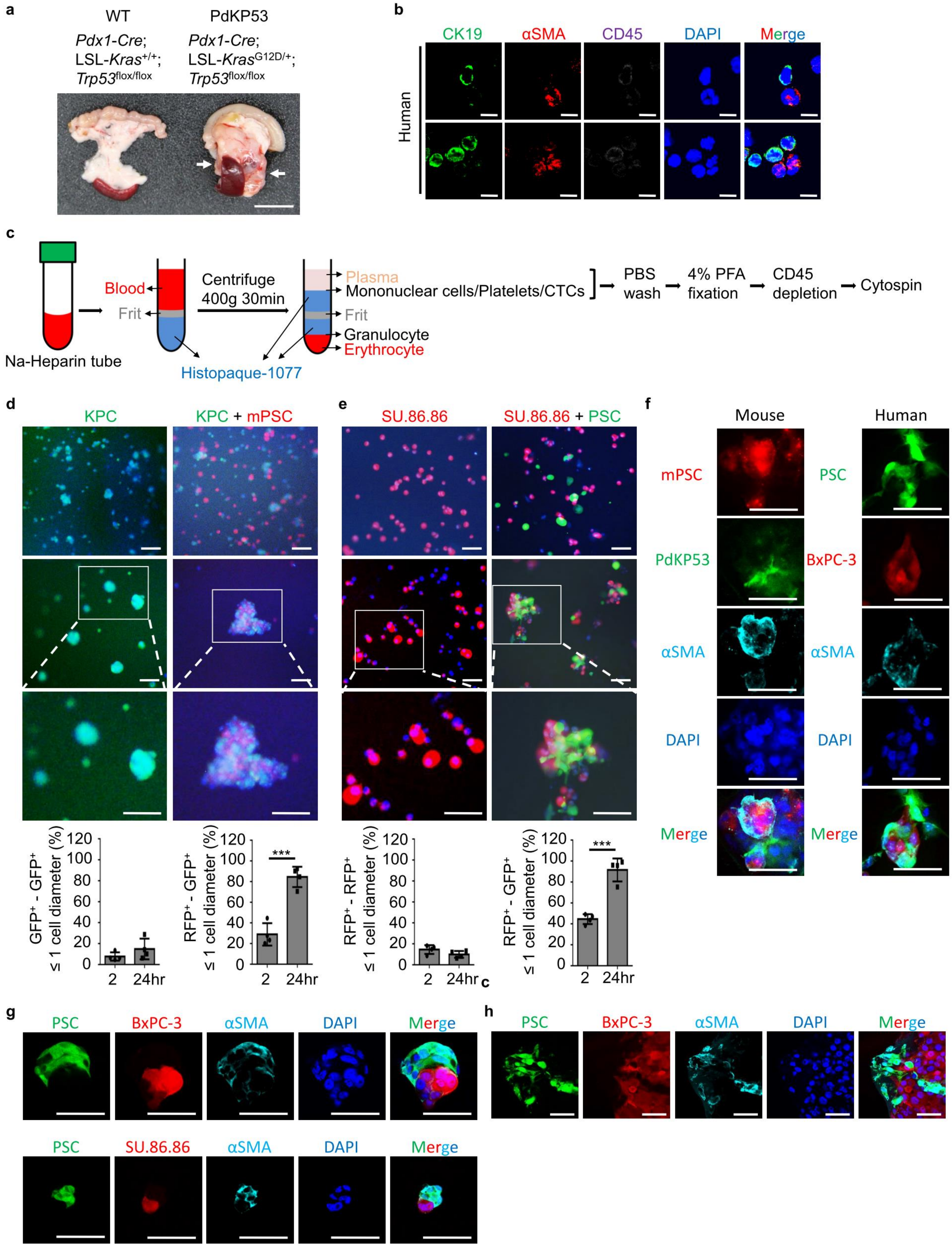

Supplemental Figure 1. αSMA$^+$ fibroblasts directly bind to tumor cells.

(a) Pancreas from 7 to 8 weeks old WT and PdKP53 mice. Scale bar, 1 cm. **(b)** The schematic procedure to isolate circulating tumor microemboli for immunofluorescence (IF) staining. Tumor cells were enriched by Accuspin System-Histopaque-1077 tubes and depletion of CD45$^+$ cells. Cells were deposited onto glass slides by Cytospin centrifuge for IF staining. Frit: Porous high density polyethylene barrier. **(c)** Representative IF images of CK19/αSMA/CD45/DAPI expression in circulating tumor-fibroblast clusters in PDAC patients. Scale bar, 10 μm. **(d and e)** 3D-Matrigel mono-culture or co-culture assay of **(d)** mouse pancreatic stellate cells (mPSC-RFP) and mouse pancreatic cancer cells (KPC-GFP) and **(e)** human pancreatic stellate cells (PSC-GFP) and human pancreatic cancer cells (SU.86.86-RFP). Upper panel, representative images. Scale bar, 100 μm. Lower panel**,** after 2 hrs and 24 hrs, the percentage of RFP$^+$-RFP$^+$ or RFP$^+$-GFP$^+$ cells with distance ≤ 1 cell diameter were determined by Imaris software at indicated time points. Values were presented as mean ± SD (n = 4). ***$p < 0.001$ (two-tailed t-test). **(f-h)** Immunofluorescence staining of αSMA in **(f)** fibroblasts (mPSC-RFP and PSC-GFP) and cancer cells (PdKP53-GFP and BxPC-3-RFP) in 3D-based tumor-fibroblast aggregates, **(g)** fibroblasts (PSC-GFP) and cancer cells (BxPC-3-RFP and SU.86.86-RFP) in tumor-fibroblast spheroids, and **(h)** fibroblasts (PSC-GFP) and cancer cells (BxPC-3-RFP) in spheroid invasion front measured by confocal microscope. Scale bar, 50 μm.

Supplemental Figure 2

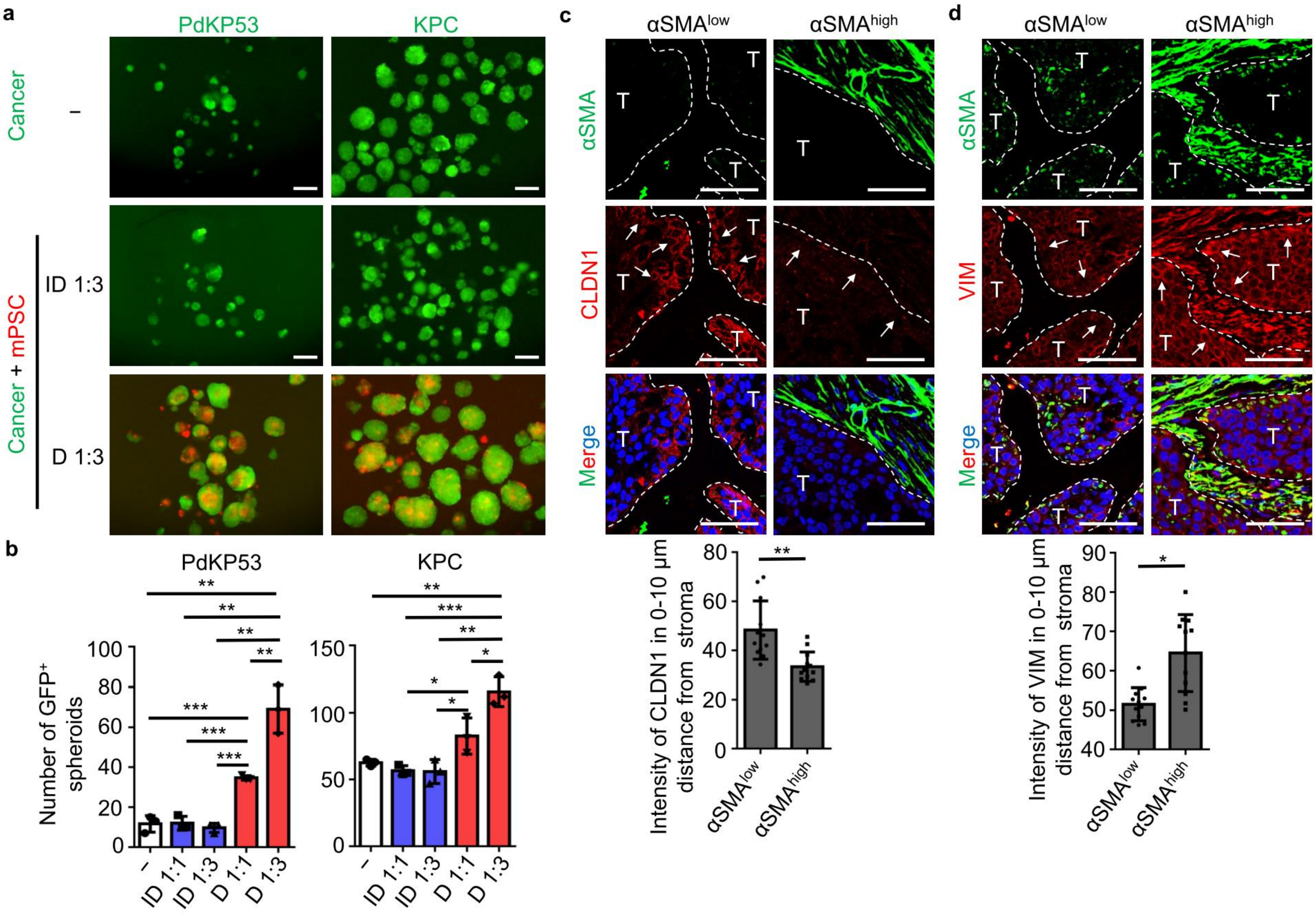

Supplemental Figure 2. Direct contact with fibroblasts promotes EMT of tumor cells.
(a and b) Sphere-formation assay of mono-cultured, indirect-cocultured (ID), and direct-cocultured (D) tumor cells (PdkP53-GFP and KPC-GFP) with fibroblast-RFP cells. Representative images **(a)** and quantification results of GFP$^+$ spheroids **(b)**. Scale bar, 200 μm. Values were presented as mean ± SD (n = 4). **(c and d)** IHC images of αSMA/CLDN1/DAPI or αSMA/VIM/DAPI expression in xenograft tumors of BxPC-3 cells co-injected with PSCs in NSG mice. Upper panel, representative images. Tumor regions were marked by T. Scale bar, 50 μm. Lower panel, the expression levels of CLDN1 and VIM in tumor cells were compared between regions with low and high tumor adjacent αSMA$^+$ fibroblasts. Values were presented as mean ± SD (n = 12). *$p < 0.05$, **$p < 0.01$, ***$p < 0.001$ (two-tailed t-test).

Supplemental Figure 3

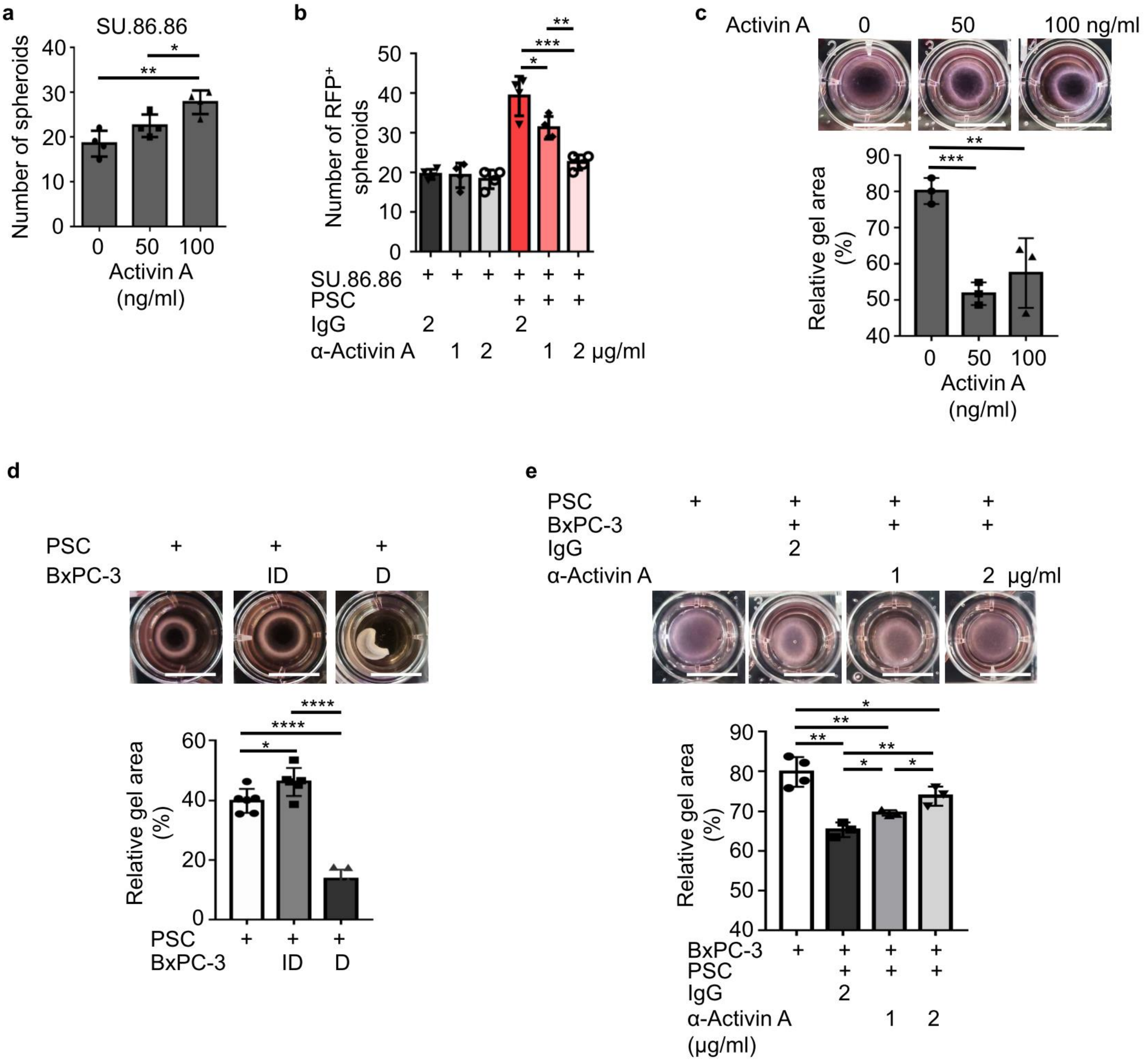

Supplemental Figure 3. Direct tumor-fibroblast contact triggers activin A secretion to enhance tumorsphere formation and myofibroblast contractility.

(a) Sphere-formation assay of SU.86.86 cells treated with recombinant human activin A compared to untreated cells. Values were presented as mean ± SD (n = 4). **(b)** Sphere-formation assay of SU.86.86-RFP cells in mono-cultured and direct-cocultured conditions treated with IgG (2 µg/ml) or anti-activin A-neutralizing antibody (1 and 2 µg/ml). Values were presented as mean ± SD (n = 4). **(c)** Collagen gel contraction assay of PSCs treated with recombinant human activin A (50 and 100 ng/ml) compared to untreated cells. Upper panel, representative images. Scale bar, 1 cm. Lower panel, relative gel area was calculated by normalizing gel area at 72 hrs to gel area at 1hr. Values were presented as mean ± SD (n = 3). **(d)** Collagen gel contraction assay of PSCs in mono-cultured, indirect-cocultured (ID), and direct-cocultured (D) conditions with tumor cells (BxPC-3). Upper panel, representative images. Scale bar, 1 cm. Lower panel, relative gel area was calculated by normalizing gel area at 72 hrs to gel area at 1hr. Values were presented as mean ± SD (n = 6). **(e)** Collagen gel contraction assay of PSCs in mono-cultured and direct-cocultured conditions treated with IgG (2µg/ml) or anti-activin A-neutralizing antibody (1 and 2µg/ml). Upper panel, representative images. Scale bar, 1 cm. Lower panel, relative gel area was calculated by normalizing gel area at 72 hrs to gel area at 1 hr. Values were presented as mean ± SD (n = 3). *$p < 0.05$, **$p < 0.01$, ***$p < 0.001$, ****$p < 0.0001$ (two-tailed t-test).

Supplemental Figure 4

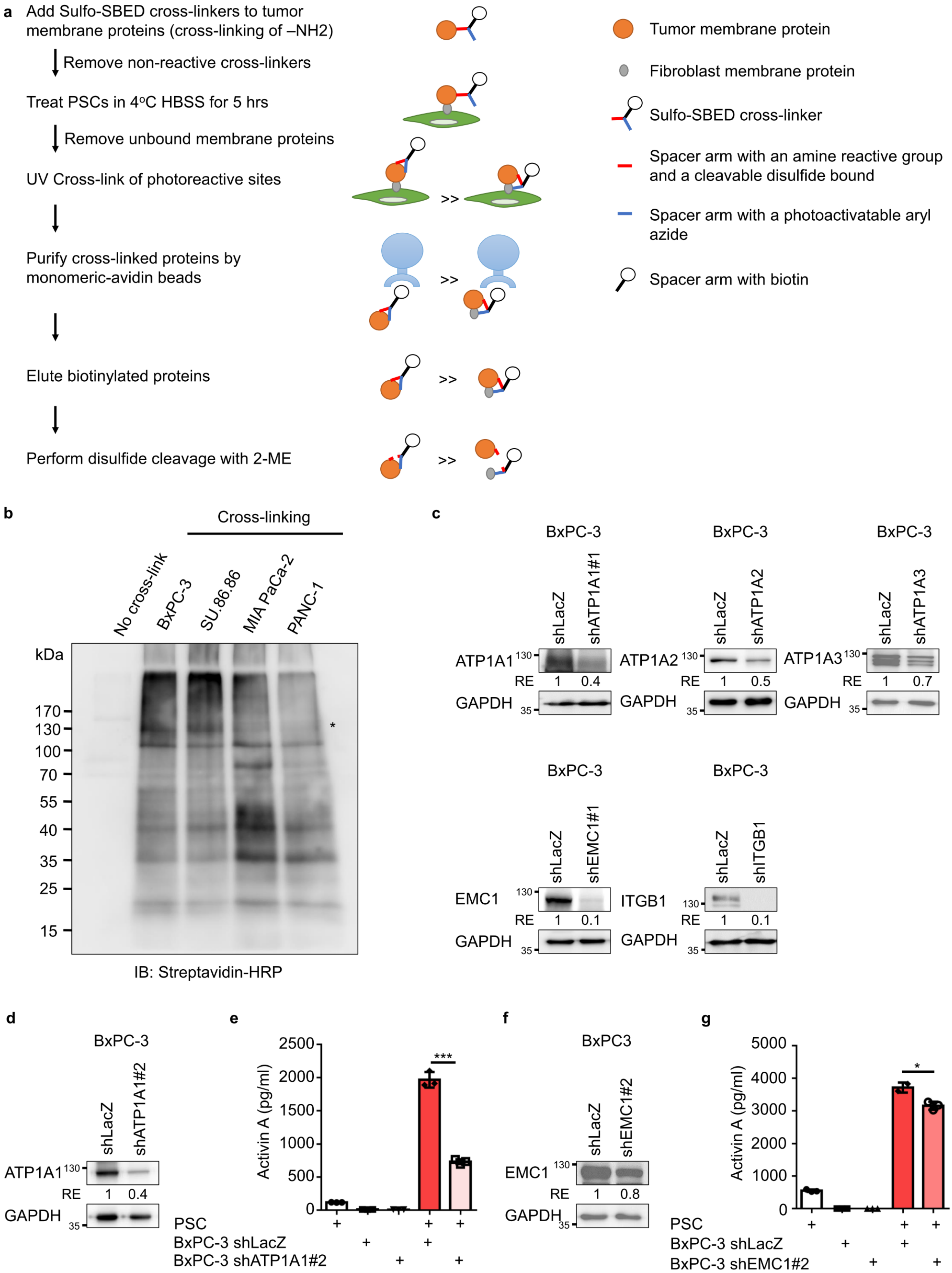

Supplemental Figure 4. Cross-linking of membrane proteins identified ATP1A1 for contact-mediated activin A secretion.

(a) The schematic procedure of membrane protein identification. Sulfo-SBED is a trifunctional crosslinker with an amine reactive group separated by a disulfide bond from a UV-activated aryl azide and a biotin. Sulfo-SBED crosslinkers bound to tumor membrane proteins through amine reactive groups with cleavable disulfide bond (red bond). After removal of unbound crosslinkers, membrane proteins with Sulfo-SBED were added to PSCs for 5 hrs to enable sufficient protein binding. Unbound proteins were removed and cells were exposed to UV lamp to activate aryl azide group for crosslinking (blue bond). In the end, crosslinked proteins were purified by monomeric-avidin beads and eluted for western blotting analysis and mass spectrometry analysis. **(b)** Western blotting analysis of no cross-linking control and cross-linked membrane protein complexes with streptavidin-HRP under reducing condition. Protein bands (*) from five individual lanes were cutted for protein ID identification by mass spectrometry. **(c)** Western blotting analysis of proteins harvested from BxPC-3 cells stably expressing lentiviral-based $LacZ^{shRNA}$, $ATP1A1^{shRNA\ clone\ \#1}$, $ATP1A2^{shRNA}$, $ATP1A3^{shRNA}$, $EMC1^{shRNA\ clone\ \#1}$, and $ITGB1^{shRNA}$. **(d)** Western blotting analysis of proteins harvested from BxPC-3 cells stably expressing lentiviral-based $LacZ^{shRNA}$ and $ATP1A1^{shRNA\ clone\ \#2}$. **(e)** ELISA analysis of activin A in mono-cultured and direct-cocultured fibroblasts with BxPC-3 cells stably expressing lentiviral-based $LacZ^{shRNA}$ and $ATP1A1^{clone\ \#2\ shRNA}$. Values were presented as mean ± SD (n = 3). **(f)** Western blotting analysis of proteins harvested from BxPC-3 cells stably expressing lentiviral-based $LacZ^{shRNA}$ and $EMC1^{shRNA\ clone\ \#2}$. **(g)** ELISA analysis of activin A in mono-cultured and direct-cocultured fibroblasts with BxPC-3 cells stably expressing lentiviral-based $LacZ^{shRNA}$ and $EMC1^{shRNA\ clone\ \#2}$. Values were presented as mean ± SD (n = 3). *$p < 0.05$, ***$p < 0.001$ (two-tailed t-test).

Supplemental Figure 5

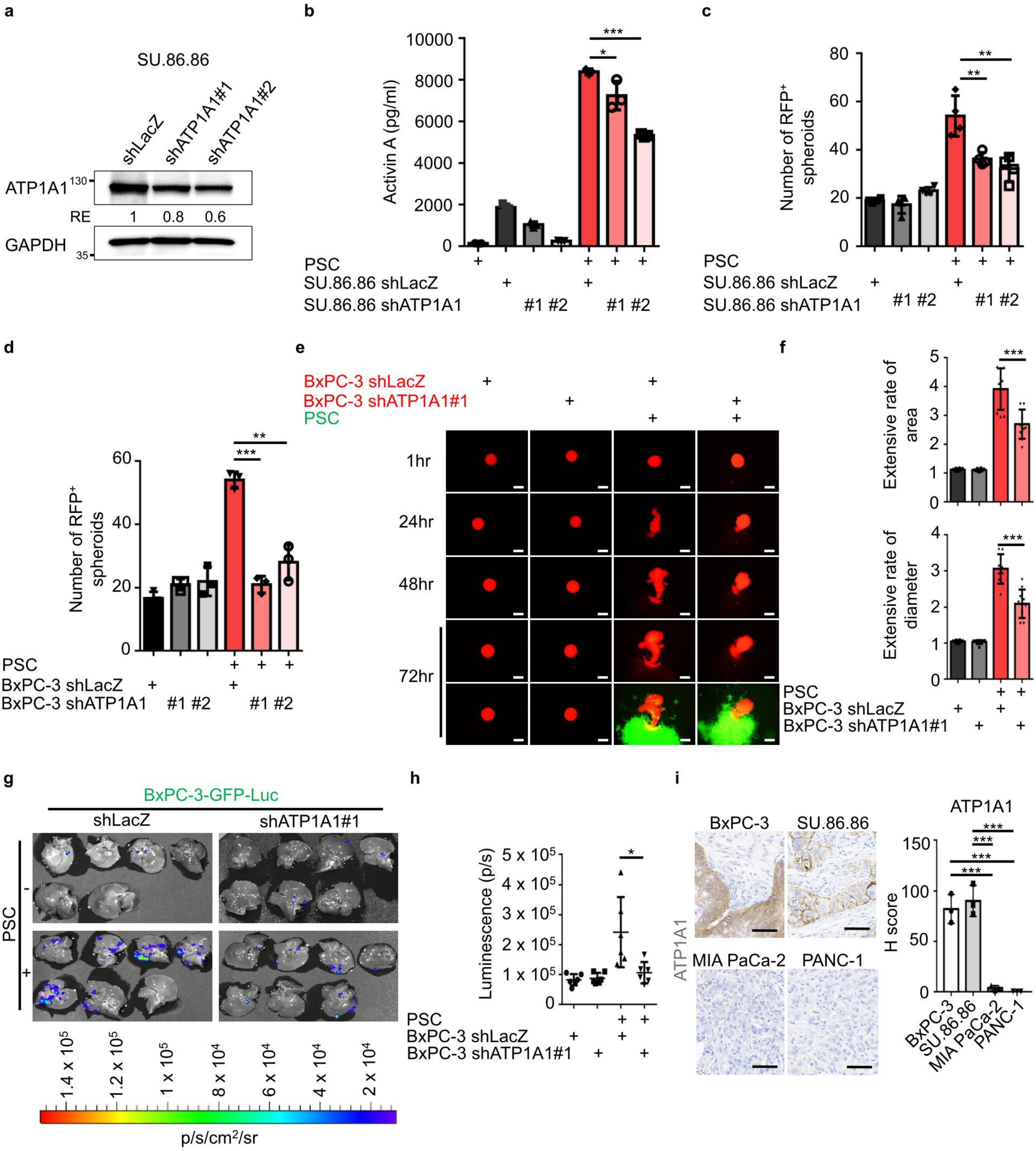

Supplemental Figure 5. ATP1A1 overexpressed in tumor cells regulates contact-mediated activin A secretion, sphere formation, invasion, and liver colonization.

(a) Western blotting analysis of proteins harvested from SU.86.86 cells stably expressing lentiviral-based $LacZ^{shRNA}$, $ATP1A1^{shRNA\ clone\ \#1}$, and $ATP1A1^{shRNA\ clone\ \#2}$. **(b)** ELISA analysis of activin A in mono-cultured and direct-cocultured fibroblasts with SU.86.86 cells stably expressing lentiviral-based $LacZ^{shRNA}$, $ATP1A1^{shRNA\ clone\ \#1}$, and $ATP1A1^{shRNA\ clone\ \#2}$. Values were presented as mean ± SD (n = 3). **(c)** Sphere-formation assay of SU.86.86 cells stably expressing lentiviral-based $LacZ^{shRNA}$, $ATP1A1^{shRNA\ clone\ \#1}$, and $ATP1A1^{shRNA\ clone\ \#2}$ to perform mono- or direct-cocultured with fibroblasts. Values were presented as mean ± SD (n = 4). **(d)** Sphere-formation assay of BxPC-3 cells stably expressing lentiviral-based $LacZ^{shRNA}$ or $ATP1A1^{shRNA\ clone\ \#1}$, and $ATP1A1^{shRNA\ clone\ \#2}$ to perform mono- or direct-cocultured with fibroblasts. Values were presented as mean ± SD (n = 3). **(e and f)** 3D spheroid invasion assay of BxPC-3-RFP spheroids stably expressing lentiviral-based $LacZ^{shRNA}$ or $ATP1A1^{shRNA\ clone\ \#1}$ in mono-cultured and direct-cocultured conditions with PSC-GFP. **(e)** Representative images. Scale bar, 200 μm. **(f)** Extensive rate of each tumor spheroid was calculated by normalizing RFP^+ area at 72 hrs to RFP^+ area at 1hr (upper panel) and by normalizing RFP^+ maximal diameter at 72 hrs to RFP^+ maximal diameter at 1hr (lower panel). Values were presented as mean ± SD (n = 10). **(g and h)** BxPC-3-GFP-Luc cells stably expressing lentiviral-based $LacZ^{shRNA}$ or $ATP1A1^{shRNA\ clone\#1}$ with and without direct contact with fibroblasts were injected into spleen of C57BL/6 mice. Liver metastasis was assessed by IVIS bioluminescent analysis (Lumina, Perkin Elmer) after 2 weeks of splenic injection. **(g)** Representative bioluminescence images of liver metastasis. **(h)** The bioluminescent signal (pseudocolor) was recorded as photons per second (p/s). Values were presented as mean ± SD (n = 6). **(i)** ATP1A1 expression in tumor xenografts of BxPC-3, SU.86.86, MIA PaCa-2, and PANC1 cells co-injection with pancreatic stellate cells. Representative IHC images (Left panel) and H score (Right panel). Scale bar, 60 μm. Values were presented as mean ± SD (n = 3). *$p < 0.05$, **$p < 0.01$, ***$p < 0.001$ (two-tailed t-test).

Supplemental Figure 6

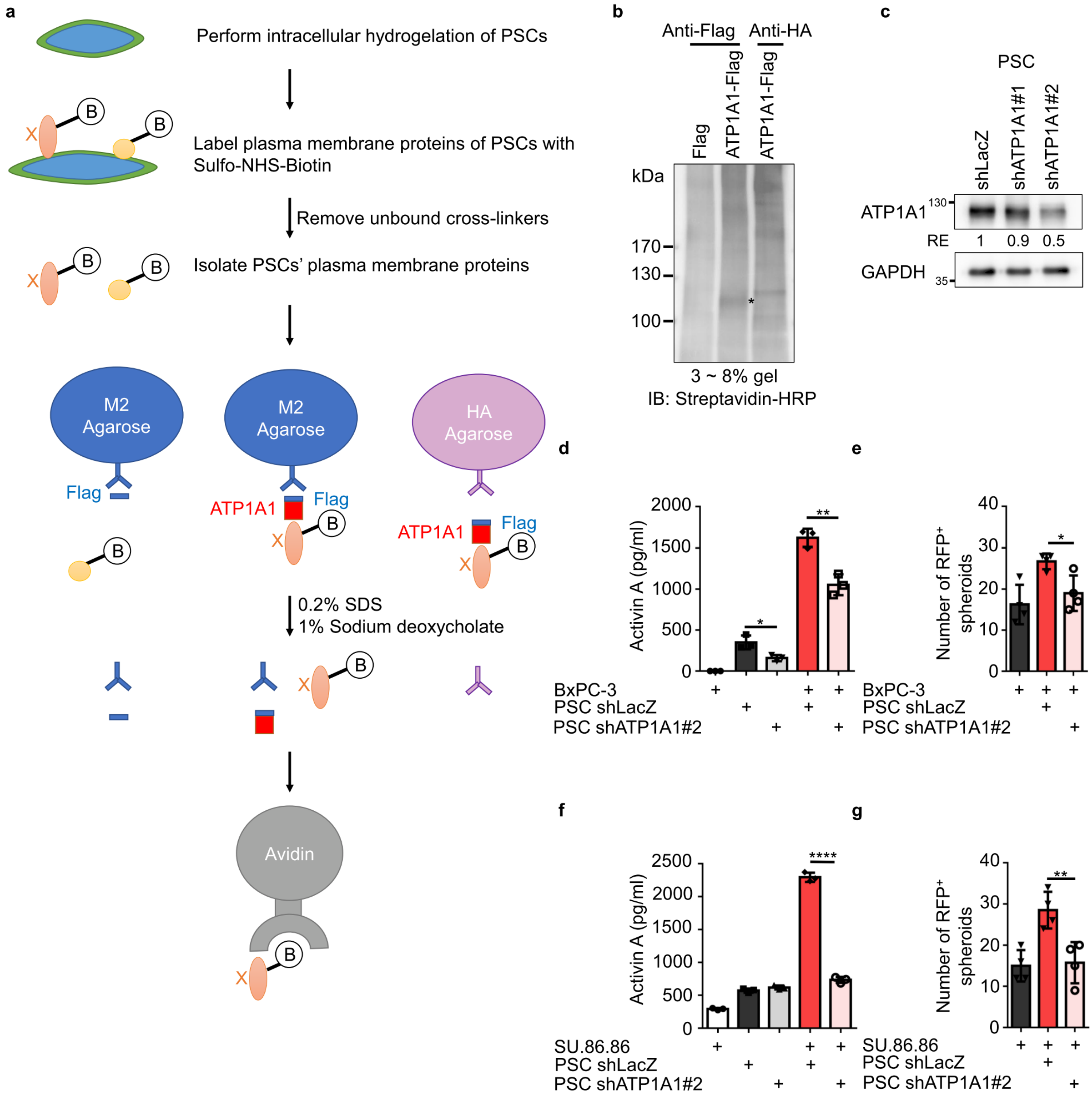

Supplemental Figure 6. Homophilic ATP1A1 interactions promote activin A secretion and sphere formation.

(a) The schematic procedure to identify ATP1A1-interating proteins. Sulfo-NHS-Biotin crosslinkers bound to plasma membrane proteins of intracellular hydrogelated fibroblasts. Biotinylated proteins of fibroblasts which bound to ATP1A1 were immunoprecipitated by ATP1A1-Flag-bound M2 agarose beads, but not by Flag-bound M2 agarose beads or anti-HA agarose beads pre-mixed with ATP1A1-Flag. Finally, biotinylated proteins were released by detergent and purified by monomeric-avidin beads. **(b)** Western blotting analysis of biotinylated fibroblast membrane proteins with streptavidin-HRP under reducing condition. Protein band (*) from three individual lanes were cut for protein ID identification by mass spectrometry. **(c)** Western blotting analysis of proteins harvested from PSCs stably expressing lentiviral-based $LacZ^{shRNA}$, $ATP1A1^{shRNA\ clone\#1}$, and $ATP1A1^{shRNA\ clone\#2}$. **(d)** ELISA analysis of activin A in mono-cultured and direct-cocultured BxPC-3 cells with fibroblasts stably expressing lentiviral-based $LacZ^{shRNA}$ and $ATP1A1^{shRNA\ clone\ \#2}$. Values were presented as mean ± SD (n = 3). **(e)** Sphere-formation assay of BxPC-3-RFP cells in mono-cultured or direct-cocultured with fibroblasts stably expressing lentiviral-based $LacZ^{shRNA}$ and $ATP1A1^{shRNA\ clone\ \#2}$. Values were presented as mean ± SD (n = 4). **(f)** ELISA analysis of activin A in mono-cultured and direct-cocultured SU.86.86 cells with fibroblasts stably expressing lentiviral-based $LacZ^{shRNA}$ and $ATP1A1^{shRNA\ clone\ \#2}$. Values were presented as mean ± SD (n = 3). **(g)** Sphere-formation assay of SU.86.86-RFP cells in mono-cultured or direct-cocultured with fibroblasts stably expressing lentiviral-based $LacZ^{shRNA}$ and $ATP1A1^{shRNA\ clone\ \#2}$. Values were presented as mean ± SD (n = 4). *$p < 0.05$, **$p < 0.01$, ****$p < 0.0001$ (two-tailed t-test).

Supplemental Figure 7

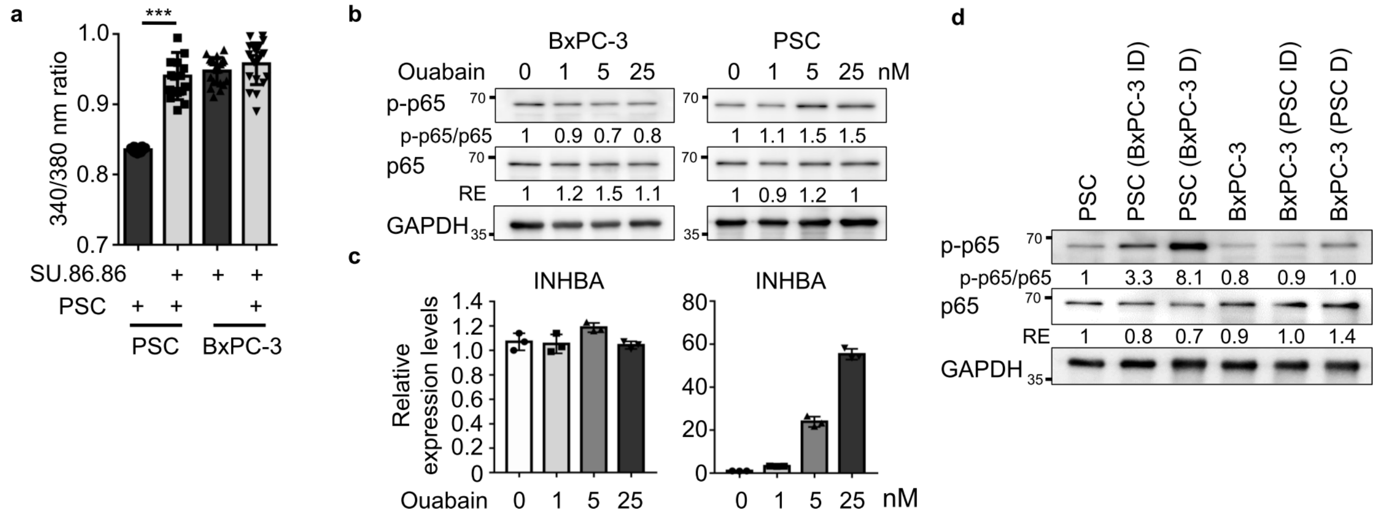

Supplemental Figure 7. Direct contact with tumor cells triggers intracellular Ca^{2+} oscillations for turning on NF-κB signaling and activin A transcription.

(a) Intracellular Ca^{2+} concentration of SU.86.86 cells and PSCs in mono-cultured or direct co-cultured conditions measured by fluorescence ratios (340/380 nm) using Fura-2/AM. Values were presented as mean ± SD (n = 20). $***p < 0.001$ (two-tailed t-test). **(b)** Western blotting analysis of proteins harvested from fibroblasts after 48 hrs of low dose Ouabain treatment (0, 1, 5, and 25 nM). **(c)** Quantitative real-time PCR analyses were performed to compare gene expression levels of INHBA in fibroblasts after 48 hrs of low dose Ouabain treatment (0, 1, 5, and 25 nM). Values were presented as mean ± SD (n = 3). **(d)** Western blotting analysis of proteins harvested from mono-, indirect-, and direct- co-cultured fibroblast-GFP and BxPC-3 cells. Fluorescence activated cell sorting (FACS) was performed to collect GFP^{+} and GFP^{-} cells after 48 hours of mono-, indirect-, or direct- co-cultured conditions.
