# Supplementary material for: Homophilic ATP1A1 binding induces activin A secretion to promote EMT of tumor cells and myofibroblast activation
Source: Nat Commun. 2022 May 26;13:2945. doi: 10.1038/s41467-022-30638-4 (PMC9135720; doi:10.1038/s41467-022-30638-4)
Supplement: Supplementary file 3 — Reporting Summary [file 41467_2022_30638_MOESM3_ESM.pdf]

nature portfolio

Corresponding author(s): Chun-Mei Hu
Wen-Hwa Lee

Last updated by author(s): Apr 7, 2022

Reporting Summary

Nature Portfolio wishes to improve the reproducibility of the work that we publish. This form provides structure for consistency and transparency in reporting. For further information on Nature Portfolio policies, see our Editorial Policies and the Editorial Policy Checklist.

Statistics

For all statistical analyses, confirm that the following items are present in the figure legend, table legend, main text, or Methods section.

n/a	Confirmed	
☐	☒	The exact sample size (*n*) for each experimental group/condition, given as a discrete number and unit of measurement
☐	☒	A statement on whether measurements were taken from distinct samples or whether the same sample was measured repeatedly
☐	☒	The statistical test(s) used AND whether they are one- or two-sided<br>*Only common tests should be described solely by name; describe more complex techniques in the Methods section.*
☒	☐	A description of all covariates tested
☐	☒	A description of any assumptions or corrections, such as tests of normality and adjustment for multiple comparisons
☐	☒	A full description of the statistical parameters including central tendency (e.g. means) or other basic estimates (e.g. regression coefficient) AND variation (e.g. standard deviation) or associated estimates of uncertainty (e.g. confidence intervals)
☐	☒	For null hypothesis testing, the test statistic (e.g. *F*, *t*, *r*) with confidence intervals, effect sizes, degrees of freedom and *P* value noted<br>*Give P values as exact values whenever suitable.*
☒	☐	For Bayesian analysis, information on the choice of priors and Markov chain Monte Carlo settings
☒	☐	For hierarchical and complex designs, identification of the appropriate level for tests and full reporting of outcomes
☐	☒	Estimates of effect sizes (e.g. Cohen's *d*, Pearson's *r*), indicating how they were calculated

Our web collection on statistics for biologists contains articles on many of the points above.

Software and code

Policy information about availability of computer code

Data collection	Confocal data was collected using LAS X (version 3.7.4.23463). Fluorescent images of 3D-Matrigel co-culture assay, tumor sphere formation assay, and 3D spheroid invasion assay were captured by Olympus DPController. Chemiluminescent signal of western bolt was detected by Biorad ChemiDoc MP imaging System. qPCR data was collected using QuantStudio Design & Analysis Software (version 1.5.1). The bioluminescent signal of tumor cells in mice was measured by Living image (version 3.2) for IVIS Spectrum platform. Calcium imaging was captured by MetaFluor software (Version 7.0).
Data analysis	RNA-Seq reads were processed using CLC Genomics Workbench v.10 software. KEGG database was used in pathway enrichment analysis and the pathway map was plotted by pathview package in R (version 4.0.3). Gene ontology (GO) enrichment was analyze by GO-TermFinder. Raw MS files were analyzed by Maxquant (version 1.6.14). GraphPad PRISM (version 7.0) was used for statistical analysis. Medcalc (version 11.5.0.0) was used for survival analysis. Cell-cell distance in 3D-Matrigel co-culture assay was analyzed by Imaris x64 (version 9.3.0). H score of IHC images was calculated by Aperio ImageScope (version 12.4.0.5043).

For manuscripts utilizing custom algorithms or software that are central to the research but not yet described in published literature, software must be made available to editors and reviewers. We strongly encourage code deposition in a community repository (e.g. GitHub). See the Nature Portfolio guidelines for submitting code & software for further information.

Data

Policy information about availability of data

All manuscripts must include a data availability statement. This statement should provide the following information, where applicable:

- Accession codes, unique identifiers, or web links for publicly available datasets
- A description of any restrictions on data availability
- For clinical datasets or third party data, please ensure that the statement adheres to our policy

The mass spectrometry proteomics data have been deposited to the ProteomeXchange Consortium via the PRIDE partner repository with the dataset identifier PXD029467 and 10.6019/PXD029467". RNA-seq data that support the findings of this study have been deposited in the Sequence Read Archive (SRA) under accession codes from SRR16629553 to SRR16629567.

Field-specific reporting

Please select the one below that is the best fit for your research. If you are not sure, read the appropriate sections before making your selection.

☒ Life sciences ☐ Behavioural & social sciences ☐ Ecological, evolutionary & environmental sciences

For a reference copy of the document with all sections, see nature.com/documents/nr-reporting-summary-flat.pdf

Life sciences study design

All studies must disclose on these points even when the disclosure is negative.

Sample size	Sample size for each experiment is indicated in all figure legends. Sample sizes were chosen based on standards of the field. In most experiments, at least three biological repeats were performed. The number of patients included in the study was determined by the availability of patients' specimens.
Data exclusions	No data were excluded from analysis.
Replication	Tissue culture and animal experiments were repeated at least twice independently. All attempts at replication were successful. Replication in LC-MS/MS analysis and RNA-seq analysis is not applicable as it is of an exploratory character.
Randomization	All mice and cells were kept in the same conditions and randomly selected. For immunohistochemistry analysis, tissue specimens were acquired from the National Taiwan University Hospital (NTUH), which randomly selected pancreatic cancer patients.
Blinding	This study was not blinding. Experimental design and data analysis were performed by the same person.

Reporting for specific materials, systems and methods

We require information from authors about some types of materials, experimental systems and methods used in many studies. Here, indicate whether each material, system or method listed is relevant to your study. If you are not sure if a list item applies to your research, read the appropriate section before selecting a response.

Materials & experimental systems

n/a	Involved in the study	
☐	☒	Antibodies
☐	☒	Eukaryotic cell lines
☒	☐	Palaeontology and archaeology
☐	☒	Animals and other organisms
☐	☒	Human research participants
☒	☐	Clinical data
☒	☐	Dual use research of concern

Methods

n/a	Involved in the study	
☒	☐	ChIP-seq
☒	☐	Flow cytometry
☒	☐	MRI-based neuroimaging

Antibodies

Antibodies used	anti-αSMA (Mouse monoclonal [1A4], 1:200 for immunostaining, 1:1000 for immunoblotting, ab7817, abcam); anti-αSMA-488 (Mouse monoclonal [1A4], 1:100 for immunostaining, 53-9760-82, Invitrogen); anti-αSMA (rabbit polyclonal, 1:400 for immunostaining, NB600-531, Novus biological); anti-CK19 (rabbit polyclonal, 1:200 for immunostaining, GTX112666, Genetex); anti-CK19 (Mouse monoclonal [BA-17], 1:150 for immunostaining, GTX27755, Genetex); anti-Vimentin (rabbit polyclonal, 1:500 for immunostaining, 10366-1-AP, Proteintech); anti-Vimentin (rabbit monoclonal [D21H3], 1:1000 for immunoblotting, 5741, Cell Signaling); anti-Claudin1 (1:100 for immunostaining; 51-9000, Invitrogen); anti-ATP1A1 (Rabbit monoclonal [HL114], 1:200 for immunostaining, GTX635461, Genetex); anti-ATP1A1 (rabbit polyclonal, 1:50 for immunostaining, 1:10000 for immunoblotting,

14418-1-AP, Proteintech); anti-EpCAM (Mouse monoclonal [G8.8], 1:200 for immunostaining, 14-5791-85, eBioscience); anti-PDGFRα (Mouse monoclonal [16A1], 1:200 for immunostaining, ab48202, abcam); anti-CD45 (rat monoclonal [RA3-6B2], 1:100 for immunostaining, 14-0452-85, eBioscience); anti-Activin A (rabbit polyclonal, 1:20 for immunostaining, 10651-1-AP, Proteintech); anti-Activin A (mouse monoclonal [A15095A], 1~4μg/ml for neutralization, 693604, Biolegend); anti-Claudin1(CLDN1)(Rabbit polyclonal, 1:1000 for immunoblotting, 4933, Cell Signaling); anti-Snail (Rabbit monoclonal [C15D3], 1:1000 for immunoblotting, 3879, Cell Signaling); anti-Twist1 (Rabbit oligoclonal, 1:1000 for immunoblotting, 711565, Invitrogen); anti-ZEB1 (Rabbit monoclonal [D80D3], 1:1000 for immunoblotting, 3396, Cell Signaling); anti-ZEB2 (Rabbit monoclonal [E6U7Z], 1:1000 for immunoblotting, 97885, Cell Signaling); anti-ATP1A2 (Rabbit polyclonal, 1:1000 for immunoblotting, 16836-1-AP, Proteintech); anti-ATP1A3 (Rabbit polyclonal, 1:1000 for immunoblotting, 28030-1-AP, Proteintech); anti-EMC1 (Rabbit polyclonal, 1:1000 for immunoblotting, 2~4μg/ml for neutralization, GTX119884, Genetex); anti-ITGB1 (Rabbit polyclonal, 1:1000 for immunoblotting, 12594-1-AP, Proteintech); anti-GAPDH (mouse monoclonal [GT239], 1:10000 for immunoblotting, GTX627408, Genetex); anti-Collagen1 (Rabbit polyclonal, 1:1000 for immunoblotting, ab34710, abcam); anti-Flag (mouse monoclonal [M2], 1:1000 for immunoblotting, F3165, Sigma); anti-Flotillin1 (Rabbit monoclonal [EPR6041], 1:1000 for immunoblotting, ab133497, abcam); anti-p-p65 (S536) (Rabbit monoclonal [93H1], 1:1000 for immunoblotting, 3033, Cell Signaling); anti-p65 (Rabbit polyclonal, 1:1000 for immunoblotting, 3034, Cell Signaling). Fluorescent secondary antibodies (1:200 for immunostaining, Donkey anti-mouse Alexa Fluor 488 (A21202), Donkey anti-rat Alexa Fluor 647 (A78947), and Donkey anti-rabbit Alexa Fluor 594 (A21207)) were purchased from Invitrogen.

Validation

anti-αSMA (Mouse monoclonal [1A4], ab7817, abcam) was validated by 767 refs as reported by the manufacturer (https://www.abcam.com/alpha-smooth-muscle-actin-antibody-1a4-ab7817.html). This antibody is validated for IF, IHC, and WB in human.
anti-αSMA (rabbit polyclonal, NB600-531, Novus biological) was validated by 50 refs as reported by the manufacturer (https://www.novusbio.com/products/alpha-smooth-muscle-actin-antibody_nb600-531#PublicationSection). This antibody is validated for IF, IHC, and WB in human and mouse.
anti-CK19 (rabbit polyclonal, GTX112666, Genetex) was previously validated (Huang HJ et al., Mol Biosyst 2014). This antibody is validated for WB, IF, and IHC in human and mouse.
anti-CK19 (Mouse monoclonal [BA-17], GTX27755, Genetex) was validated by 3 refs as reported by the manufacturer (https://www.genetex.com/Product/Detail/Cytokeratin-19-antibody-BA-17/GTX27755#references). This antibody is validated for WB, IF, and IHC in human and mouse.
anti-Vimentin (rabbit polyclonal, 10366-1-AP, Proteintech) was validated by 691 refs as reported by the manufacturer (https://www.ptglab.com/products/VIM-Antibody-10366-1-AP.htm). This antibody is validated for WB, IF, and IHC in human and mouse.
anti-Vimentin (rabbit monoclonal [D21H3], 5741, Cell Signaling) was validated by 1315 refs as reported by the manufacturer (https://www.cellsignal.com/products/primary-antibodies/vimentin-d21h3-xp-rabbit-mab/5741). This antibody is validated for WB, IF, and IHC in human and mouse.
anti-ATP1A1 (rabbit polyclonal, 14418-1-AP, Proteintech) was validated by 68 refs as reported by the manufacturer (https://www.ptglab.com/products/ATP1A1-Antibody-14418-1-AP.htm). This antibody is validated for WB, IF, IP, and IHC in human and mouse.
anti-EpCAM (Mouse monoclonal [G8.8], 14-5791-85, ebioscience) was validated by 77 refs as reported by the manufacturer (https://www.thermofisher.com/antibody/product/CD326-EpCAM-Antibody-clone-G8-8-Monoclonal/14-5791-85). This antibody is validated for FACS in mouse.
anti-CD45 (rat monoclonal [RA3-6B2], 14-0452-85, eBioscience) as validated by 264 refs as reported by the manufacturer (https://www.thermofisher.com/antibody/product/CD45R-B220-Antibody-clone-RA3-6B2-Monoclonal/14-0452-85). This antibody is validated for IF and FACS in human and mouse.
anti-Activin A (rabbit polyclonal, 10651-1-AP, Proteintech) was validated by 10 refs as reported by the manufacturer (https://www.ptglab.com/products/INHBA-Antibody-10651-1-AP.htm). This antibody is validated for WB, IF, and IHC in human and mouse.
anti-Claudin1 (Rabbit polyclonal, 4933, Cell Signaling) was validated by 34 refs as reported by the manufacturer (https://www.cellsignal.com/products/primary-antibodies/claudin-1-antibody/4933). This antibody is validated for WB in human.
anti-Snail (Rabbit monoclonal [C15D3], 3879, Cell Signaling) was validated by 669 refs as reported by the manufacturer (https://www.cellsignal.com/products/primary-antibodies/snail-c15d3-rabbit-mab/3879). This antibody is validated for WB in human.
anti-ZEB1 (Rabbit monoclonal [D80D3], 3396, Cell Signaling) was validated by 256 refs as reported by the manufacturer (https://www.cellsignal.com/products/primary-antibodies/zeb1-d80d3-rabbit-mab/3396). This antibody is validated for WB in human.
anti-ZEB2 (Rabbit monoclonal [E6U7Z], 97885, Cell Signaling) was previously validated (Hui Yao, et. al., Int J Oncol 2020). This antibody is validated for WB in human.
anti-ATP1A2 (Rabbit polyclonal, 16836-1-AP, Proteintech) was validated by 12 refs as reported by the manufacturer (https://www.ptglab.com/products/ATP1A2-Antibody-16836-1-AP.htm). This antibody is validated for WB, IF, and IHC in human and mouse.
anti-EMC1 (Rabbit polyclonal, GTX119884, Genetex) was previously validated (Janer A et al., EMBO Mol Med. 2016). This antibody is validated for WB and IHC in human.
anti-ITGB1 (Rabbit polyclonal, 12594-1-AP, Proteintech) was validated by 26 refs as reported by the manufacturer (https://www.ptglab.com/products/ITGB1-Antibody-12594-1-AP.htm). This antibody is validated for WB, IF, and IHC in human and mouse.
anti-GAPDH (mouse monoclonal [GT239], GTX627408, Genetex) was validated by 314 refs as reported by the manufacturer (https://www.genetex.com/Product/Detail/GAPDH-antibody-GT239/GTX627408#references). This antibody is validated for WB, IF, and IHC in human and mouse.
anti-Collagen1 (Rabbit polyclonal, ab34710, abcam) was validated by 1318 refs as reported by the manufacturer (https://www.abcam.com/collagen-i-antibody-ab34710.html). This antibody is validated for WB in human.
anti-Flotillin1 (Rabbit monoclonal [EPR6041], ab133497, abcam) was validated by 31 refs as reported by the manufacturer (https://www.abcam.com/flotillin-1-antibody-epr6041-ab133497.html). This antibody is validated for WB, IF, and IHC in human and mouse.
anti-p-p65 (S536) (Rabbit monoclonal [93H1], 3033, Cell Signaling) was validated by 2195 refs as reported by the manufacturer (https://www.cellsignal.com/products/primary-antibodies/phospho-nf-kb-p65-ser536-93h1-rabbit-mab/3033). This antibody is validated for WB and IF in human and mouse.
anti-p65 (Rabbit polyclonal, 3034, Cell Signaling) was validated by 304 refs as reported by the manufacturer (https://www.cellsignal.com/products/primary-antibodies/nf-kb-p65-antibody/3034). This antibody is validated for WB in human and mouse.

Eukaryotic cell lines

Policy information about cell lines

Cell line source(s)

HEK293T, BxPC-3, SU.86.86, HPAF-II, AsPC-1, MIA PaCa-2, and PANC-1 were obtained from ATCC. HPaSteC was from ScienCell

Cell line source(s)	Research Laboratories, inc.. Mouse primary pancreatic stellate cells were isolated from 8 week-old male C57BL/6 mice using Nycodenz gradient method. Patient-derived primary pancreatic fibroblasts (PSC-2) were isolated from human specimens with IPMN lesion and without PDAC.
Authentication	Cell lines were not authenticated.
Mycoplasma contamination	All cell lines were routinely tested for mycoplasma and contaminated cells were withdrawal. All cell lines tested negative for mycoplasma contamination.
Commonly misidentified lines (See ICLAC register)	No commonly misidentified cell lines were used in this study.

Animals and other organisms

Policy information about studies involving animals; ARRIVE guidelines recommended for reporting animal research

Laboratory animals	8-week-old female C57BL/6 mice were obtained from National Laboratory Animal Center (Taipei, Taiwan). 6 to 7-week-old male Pdx1-Cre; LSL-Kras+/+; Trp53flox/flox (WT), male Pdx1-Cre; LSL-KrasG12D/+; Trp53flox/flox (PdKP53), and 6 to 8-week-old male NOD/SCID/IL2Rγnull (NSG) mice were bred in Genomics Research Center animal facility (Taipei, Taiwan).
Wild animals	Wild animals were not involved in this study.
Field-collected samples	Field-collected samples were not involved in this study.
Ethics oversight	All animal experiments were reviewed by Institutional Animal Care and Use Committee and performed experiments according to the guidelines for laboratory animal research of Academia Sinica (Taipei, Taiwan).

Note that full information on the approval of the study protocol must also be provided in the manuscript.

Human research participants

Policy information about studies involving human research participants

Population characteristics	Patients characteristics were listed in Table 1. Based on the 7th edition of the American Joint Committee on Cancer (AJCC) criteria, PDAC patients were staged as IIA (n = 32), IIB (n = 52), and 1 unknown status. For immunohistochemistry (IHC) analysis, total patient specimens were used. For in situ mRNA hybridization, patient specimens (n = 19) without positive control signals were removed in consideration of poor RNA quality.
Recruitment	All pancreatic cancer patient data and tissue specimens were acquired from the National Taiwan University Hospital (NTUH), Taipei, Taiwan. Between 2007 and 2015, pancreatic cancer specimens were collected from 85 patients who underwent pancreaticoduodenectomy in NTUH. The patients were recruited by the surgeons involved in the study. Since surgery can only be performed in PDAC at an earlier stage, the clinical specimens were composed of stage II PDAC.
Ethics oversight	The study was approved by the Institutional Review Board of the NTUH (201303029RINC, 201411085RINB, and 201701015RINA).

Note that full information on the approval of the study protocol must also be provided in the manuscript.
